# Cognitive comorbidities in the rat pilocarpine model of epilepsy

**DOI:** 10.3389/fneur.2024.1392977

**Published:** 2024-05-30

**Authors:** Annunziata Guarino, Paola Pignata, Francesca Lovisari, Laila Asth, Michele Simonato, Marie Soukupova

**Affiliations:** ^1^Department of Neuroscience and Rehabilitation, Section of Pharmacology, University of Ferrara, Ferrara, Italy; ^2^Division of Neuroscience, IRCCS San Raffaele Scientific Institute, Milan, Italy

**Keywords:** epilepsy, pilocarpine model, cognitive abilities, comorbidity, rats

## Abstract

Patients with epilepsy are prone to cognitive decline, depression, anxiety and other behavioral disorders. Cognitive comorbidities are particularly common and well-characterized in people with temporal lobe epilepsy, while inconsistently addressed in epileptic animals. Therefore, the aim of this study was to ascertain whether there is good evidence of cognitive comorbidities in animal models of epilepsy, in particular in the rat pilocarpine model of temporal lobe epilepsy. We searched the literature published between 1990 and 2023. The association of spontaneous recurrent seizures induced by pilocarpine with cognitive alterations has been evaluated by using various tests: contextual fear conditioning (CFC), novel object recognition (NOR), radial and T-maze, Morris water maze (MWM) and their variants. Combination of results was difficult because of differences in methodological standards, in number of animals employed, and in outcome measures. Taken together, however, the analysis confirmed that pilocarpine-induced epilepsy has an effect on cognition in rats, and supports the notion that this is a valid model for assessment of cognitive temporal lobe epilepsy comorbidities in preclinical research.

## 1 Introduction

For many patients with epilepsy, a good quality of life and mental condition are as important as seizure control. In clinical practice, this interdisciplinary issue is often underappreciated and in epilepsy preclinical research the situation is not better. However, there is a notable rise of the awareness for neuropsychiatric comorbidities of epilepsy and the number of clinical (1, 2) and preclinical studies (3, 4) of comorbidities in epilepsy seems to grow over the years. Many clinical reports indicate that mental disorders are more common in patients with epilepsy than in the general population (5–8). In this regard, mood disorders are the most common, anxiety disorders the second most frequent and psychotic disorders the least frequent in people with temporal lobe epilepsy (5), psychoses being a little more widespread than personality disorders (6). Such high prevalence of psychiatric disorders is frequently demonstrated among patients with epilepsy when compared them with the general population or with individuals presenting non-epileptic neurological conditions (7), even if it may vary a lot due to differences in study methods and heterogeneity of epilepsy syndromes (8). More in detail, the estimated prevalence of depressive disorders in patients with epilepsy ranges from 13% to 35%, compared to less that 4% in the general population (9); the prevalence of anxiety disorders in people with epilepsy may reach 40%, compared to 3% in the general population (10); the prevalence of psychotic disorders is 5.6%–5.9% in people with epilepsy vs. 0.3% in people without it (6, 11). Besides mood and psychotic disorders, people with epilepsy suffer from cognitive decline and related attention deficit or learning and memory disabilities (12). The percentages are higher in older adults, in which the prevalence of cognitive disorders may reach 60% (12, 13). However, such studies typically include individuals with chronic epilepsy unresponsive to drug treatment (14, 15), which can make difficult the interpretation of the results. Attention deficit–hyperactivity disorders are observed in 28%–70% of people with epilepsy and are more common among children with epilepsy (16). The same problems, which worsen the quality of life of people with epilepsy, may be seen in the animals and appear in various preclinical reports.

Many scientific publications support the notion that an inadequate control of seizures may lead to changes in mood and cognition both in people with epilepsy and in laboratory animals (3, 17–19). In general, animal models allow the study of comorbidities without the bias of drug treatments (20–27). Between animal models useful for the study of epilepsy comorbidities, the pilocarpine model in rats is one of the most employed. This model mimics well the natural course of the temporal lobe epilepsy (28), which permits to monitor the development of psychiatric comorbidities in the different phases of the disease (i.e., the latency and chronic phase). Pilocarpine is a cholinergic agonist injected systemically or locally to induce status epilepticus (SE), which is followed by a latent period and the subsequently appearance of spontaneous recurrent seizures that mimics a focal epileptic condition similar to the human temporal lobe epilepsy (28). Besides the pilocarpine model, other animal models of acquired epilepsy like kainate, kindling, or traumatic brain injury are used to study comorbidities of epilepsy (3, 29). Absence models, like genetic generalized epilepsy with absence seizures like Genetic Absence Epilepsy Rats from Strasbourg (GAERS) or WAG/Rij rats have been employed for the same reason (29).

The term “cognition” can be defined as the mental action or process of acquiring knowledge and understanding through thought, experience, and the senses (30). To examine it in epilepsy, researchers use various behavioral tests assessing the starting point and evolving state of cognitive functions (e.g., learning, memory, and attention) in association with frequency of seizures or epileptic-like events. Different behavioral tests may provide different types of information. When employing contextual fear conditioning, for example, not only the memory of fear but also the context of experience is tested, so the results may be interpreted in terms of episodic memory on one hand and in terms of spatial memory on the other. Such multi-response testing can be useful in some cases but may also complicate the interpretation of the results. In addition, the outcome of learning/memory behavioral tests in animals will depend not only on the type of memory solicited, but also on the duration of the task, on the continuity or discontinuity of the procedures, on the duration of habituation and on many further factors (31). Behavioral tests of learning and memory are nearly always performed when studying epilepsy related cognitive dysfunction. Social behavior and social interaction tests are used less frequently (3, 29). Different learning and memory behavioral tasks may be applied separately or as a battery. When used in sequence, a logic order of tests (usually used from the least to the most stressful, in order to decrease the chance that behavioral responses are altered by prior test history) helps the researchers to better interpret their results in epileptic animals (32). A cognitive evaluation is typically run after testing locomotor activity and anxiety and before testing depression, which closes most of behavioral testing series. Concerning the cognitive ability tests only, their precise order in batteries may not be crucial, as the performance of animals in the different cognitive models seems to be independent of each other (33, 34). More important will be to compare the results obtained at the same disease stage.

**Figure 1:**
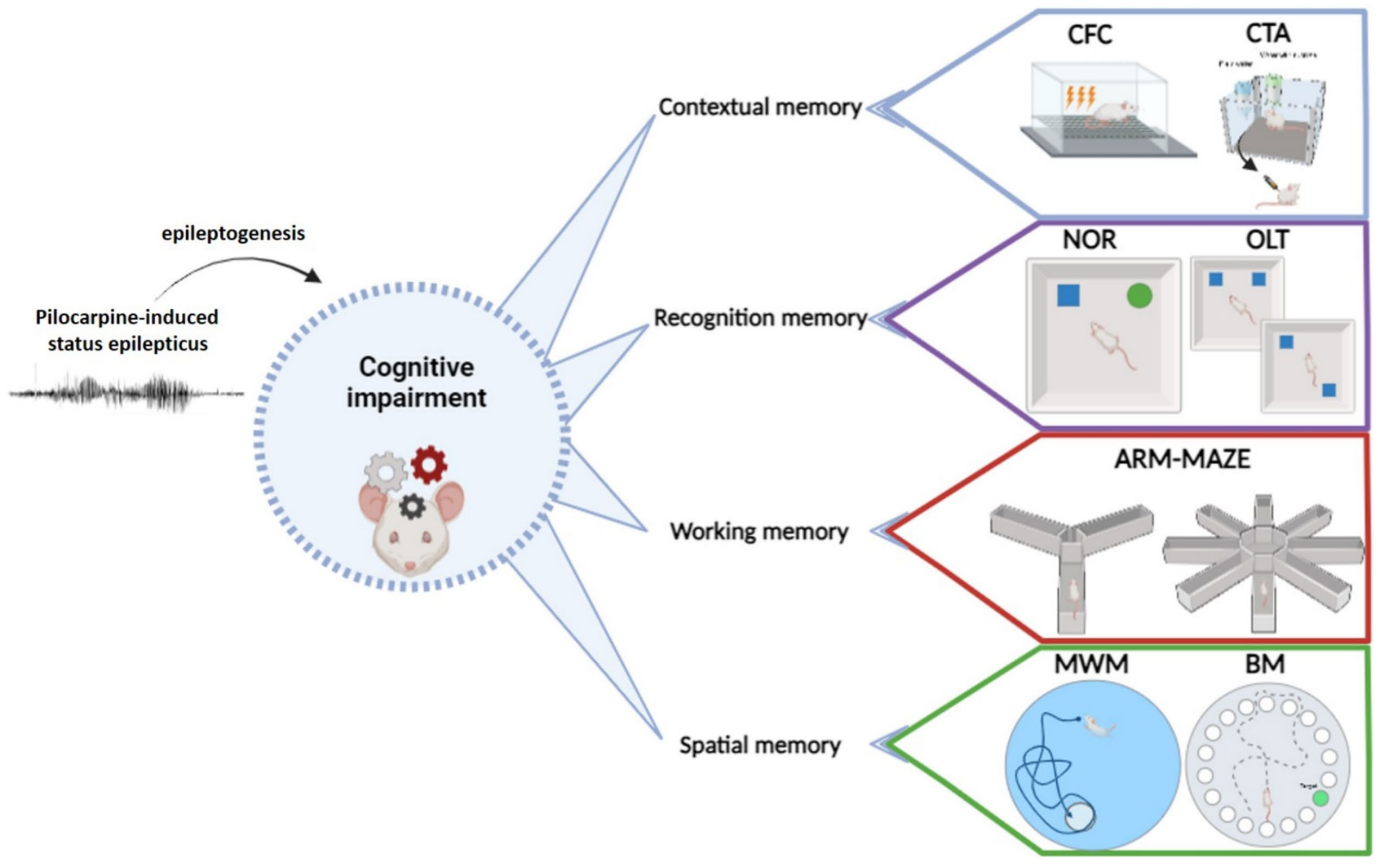
Principal behavioral tests used to evaluate cognitive abilities in epilepsy models.

Currently available tasks for studying learning and memory impairment in any neurobiological disease, epilepsy included, may be classified in different ways. One option is considering the prevailing type of memory engaged by the task (Figure 1; Table 1). In this respect (i) tests primarily measuring contextual memory include contextual and fear conditioning (CFC) and its variations, like contextual discrimination or delayed nonmatching to sample, conditioned taste aversion and social transmission of food preference test; (ii) tasks soliciting the recognition memory tests include the novel object recognition (NOR) test, the novel object preference (NOP) test and their variations; (iii) tests engaging mostly the working memory include the different mazes (T-maze, Y-maze, eight-arm radial maze); (iv) finally, tests analyzing principally the spatial memory include the Morris water maze (MWM) and the Barnes maze tests and, to some extent, the object location test (OLT) and the place preference test (PPT), in which the engagement of spatial memory is combined with contextual and working memory. It should be emphasized that this classification is not rigid. Most frequently, more than one type of memory is solicited in each cognitive task. Moreover, it is possible in many tasks to investigate only the recent, recall or remote memory. All in all, the complete spectrum of information provided by each test should be taken into account when choosing them and interpreting their results in epilepsy models. It should be also considered that, when working with epileptic animals, the situation is even more complex due to the natural progression of the disease and unpredictability of seizure occurrence.

**Table 1:** Behavioral tasks commonly used to assess cognitive comorbidities in epileptic animals.

Memory type engaged during the task	Behavioral task	Task variants	Comments
Contextual memory	Contextual and fear conditioning (CFC)	Contextual discrimination Delayed nonmatching to sample Conditioned taste aversion (CTA) Social transmission of food preference test in the group	Overlapping tests: OLT or PPT
Recognition memory	Novel object recognition (NOR) Novel object exploration (NOE) Object recognition (ORT) Spontaneous object recognition (SORT)	Novel object preference (NOP) Object location test (OLT) Place preference test (PPT)	
Working memory	Maze test	T-maze, Y-maze, eight-arm radial maze, H-maze	Overlapping tests: OLT or PPT
Spatial memory	Morris water maze (MWM) Barnes maze tests		

The details and references related to each behavioral task are provided in the text.

The aim of the present study was to overview the currently available and most utilized behavioral tests employed to study cognitive impairment in temporal lobe epilepsy, and to evaluate if there is good enough evidence for cognitive/mental comorbidities in the rat pilocarpine model. Although some of these tests may have sex specific outcomes or require sex specific settings, the large majority of the papers identified by our search employed only male rats, preventing a proper evaluation of sex-dependency.

## 2 Literature search criteria

For this review, we searched PubMed and Web of Science for any pilocarpine or lithium-pilocarpine model in rats; we selected records or original articles (reviews were excluded) written in English and published in the years 1990–2023. We used the following keywords: pilocarpine, model, rats, epilepsy, memory, fear conditioning, cued conditioning, conditional fear, contextual fear, conditioned taste aversion, Morris water maze, radial arm maze, Y-maze, T-maze, double H-maze, Barnes maze, novel object test, novel object exploration, novel object discrimination, novel placement recognition, object recognition test, spontaneous object recognition test, object recognition task, object location test, olfactory discrimination test, social recognition, hole-board task, step-down passive avoidance test, passive shock avoidance test. Studies on pups were not included. Both animals with and without electrodes implanted were considered. We included all the articles on the pilocarpine or the lithium-pilocarpine model independent of sex, pre-treatments (i.e., methyl-scopolamine), duration of SE, and of drugs used to stop SE-induced seizures (i.e., diazepam, anesthetics, anesthetics and/or analgesics). The most important inclusion criteria were the development of spontaneous recurrent seizures and their characterization (at least in terms of seizure frequency) and the specification of the time-points of behavioral testing with reference to epilepsy development and progression. All reviewed studies included non-epileptic control animals, which were compared with epileptic ones employing appropriate statistical tests. No other specific requirement regarding the study design or duration of study was set as inclusion criterion. The goal was to collect data correlating the development of epilepsy in the pilocarpine model with the assessment of memory and/or learning alterations. This search identified 104 original articles matching the inclusion criteria.

## 3 Contextual memory

### 3.1 Contextual and cued fear conditioning

Contextual and cued fear conditioning (CFC) are tests predominantly employed to assess associative learning in animals (35). The basics of CFC consist of an exposure to neutral surrounding (the context) and to a stimulus (the cue, that can be an auditory tone or a flash of light); these are then combined with an aversive unconditioned stimulus, typically a mild foot shock of a few seconds and intensity around 0.6–0.75 mA (max 1 mA in rodents). An aversive unconditioned stimulus leads to fear in animals when they are exposed to the context and cue. The outcome is a freezing behavior (a complete lack of movement except for that related to respiration); researchers mostly analyze the numbers of freezing in a defined period and/or their duration. The CFC test examines the ability of animals to anticipate unconditioned stimulus which will follow the conditioned stimulus (i.e., to learn from unpleasant experiences). The freezing behavior is generally analyzed automatically (35, 36). According to neurobehavioral findings, specific neocortical brain structures are primarily involved in fear memory, the prefrontal and the anterior cingulate cortex (36–38). Beside these, a well-established contribution of the hippocampal formation and entorhinal cortex (EC), together with inputs from basolateral amygdala (BLA), have been implicated in learning and recall of fear memory. Interestingly, the same brain structures take part in temporal lobe epilepsy circuits (39–41). Any epileptic interference with the EC-hippocampal complex, BLA or cortical memory circuits is therefore expected to influence contextual memory.

In CFC protocols, the conditioned and unconditioned stimuli are usually presented concomitantly or the unconditioned one is applied immediately after the conditioned stimulus. Such experimental flow is called “delayed conditioning.” In another type of testing, the two types of stimuli are separated by a defined time interval; such a procedure is called “trace conditioning.” Therefore, the trace conditioning differs from delay conditioning by the addition of a stimulus-free “trace” interval of seconds or minutes, separating the conditioned and unconditioned stimuli. It is believed that the longer the temporal gap in the trace protocol, the lesser the involvement of the hippocampus and the greater that of the prefrontal cortex (42). One of the most frequently employed protocols of CFC is the 2-days trial conditioning. It consists of day-1 training in a cage equipped with a sound source and with a metal grid floor. Animals are put into the cage and, after a few minutes of habituation, are exposed to an auditory stimulus of about 70–80 dB for a few tens of seconds (typically 15–30 s), before delivering a foot shock (generally 2 s of 0.6–0.75 mA) at the end of the auditory stimulus or immediately after its cessation. Such trial is then repeated for 3–5 times. After the training, the animals remain in the cage for another 1–2 min and then are put back to their home cage. In the following days, the animals are put into the same cage, and the contextual fear is evaluated by measuring the freezing response to the unpleasant context in which the animals were exposed during the training. The animals may be also put into a different context (e.g., cage with identical dimensions, but endowed with plastic floor and/or scented with an aroma) and the cue, the auditory stimulus, is then applied. Again, the freezing response is evaluated. As an alternative (similar to only context induced fear response measurement), only the cued fear may be evaluated by measuring the freezing response in the training context. Animals need more trials of training to learn the trace conditioning task. After day-1 of training, researchers may test the animals (i) the day after, in order to assess the recent memory, (ii) after 2–12 days, to test the memory consolidation process and (iii) after about 2 weeks or more, to investigate remote memory recall (memory retention). In any analyzed time point, the reduced freezing behavior indicates the impaired associative (episodic) memory, whereas an increased amount of freezing response may be associated with anxiety-like behavior (43, 44).

If used in epileptic rodents, the CFC protocol may be biased by acute seizure activity, which can resemble freezing. The spindles of spike-and-wave seizure activity may occur in parallel with freezing behavior in rodents (44, 45). Behavior arrest seizures are typical of absence epilepsy and are observed in rodent models (45, 46). In addition, the behavioral arrest associated with epileptiform spike–wave discharges has been described also in rats (47) and mice (48) exposed to pilocarpine. Considering that the CFC experiments with epileptic animals may be influenced by the occurrence of spontaneous seizures of any type (which may occur during the training, as well as at the phase of freezing response assessment), the trials should always include non-epileptic controls, and animals who experienced seizures 1–2 h prior or during the test should be excluded.

Another peculiarity of the contextual memory testing in epileptic animals relates to the learning phase. In the pilocarpine model of temporal lobe epilepsy in rats gives origin to an important hippocampal sclerosis at late phases of the disease development (28), which can lead to impairment of learning of cued fear in CFC. This does not necessarily imply that the CFC paradigm cannot be applied to epileptic animals. The different studies in adult rats treated with lithium-pilocarpine have shown similar results, i.e., an impaired contextual and cued fear memory. A small number of studies investigated fear learning or conditioning during the latency phase of the rat pilocarpine model (26, 49), without revealing any significant result. Most of the studies identified in our search, instead, evaluated fear conditioning and learning abilities in rats in the chronic phase of the disease; in particular, at about 2–3 months after SE. These studies reported a decreased contextual and/or cued fear associated with performance in pilocarpine rats, especially in advanced stages of the disease. The decrease was mainly reported in terms of lower spontaneous freezing duration in given context and/or diminished freezing time due to the tone fear conditioning cue (50, 51). Smolensky et al. (49) reported fear impairment in short term and long term memory cues only in the chronic phase (i.e., days 41–53 post SE), while such impairment was absent during latency (i.e., days 8–15 post SE). Focusing on the advanced stages of epilepsy, particular differences were consistently reported in epileptic rats that showed a significantly diminished freezing time in contextual and tone fear conditioning in comparison to controls at 2 months after pilocarpine (52) or shortly after 2 months (53). Similarly, Zubareva et al. (54) showed a contextual memory decline (decreased freezing time in the familiar cage compared to the controls) in pilocarpine rats 60–70 and 70–90 days after SE (55). Interestingly, Qiu et al. (56) found that pilocarpine rats had most severely impaired fear declarative memory at 2 months after SE, but then improved at 4 months while still performing worse than controls in CFC cues. However, one study reported an opposite result, i.e., an increased level of freezing response due to the auditory cue in delay protocol. Such a finding was observed for 4 consecutive days of training in comparison with controls at 1 month after SE (57). However, the CFC test was applied in this study to assess a single fear learning in epileptic rats at about 1 month post SE, thus differing from the others that refer to the late chronic epileptic animals at about 2–3 months post-SE. All in all, the outcome was similar: impairment of contextual memory in the chronic phase of temporal lobe epilepsy.

### 3.2 Conditioned taste aversion

Conditioned taste aversion (CTA) is another form of associative learning and may be seen as a special type of classical conditioning; the animal typically learns to associate the novel taste of a new food with subsequent illness resulting from ingestion of some nausea-inducing agent. Most animals learn the task after a single pairing of a novel taste with a nausea-inducing agent (24). Even if interesting, this test is not frequently used in animals, especially in models of temporal lobe epilepsy. Nonetheless, a few studies in the pilocarpine or Li-pilocarpine models indicate that epileptic rats acquire a weak CTA when compared to their controls, which on the other hand learn to avoid conditioned intake very early. In particular, at about 1 month after SE, pilocarpine and Li-pilocarpine treated rats displayed learning deficits and poorer performance in CTA tasks with respect to controls (58). A deterioration of CTA memory has been also observed in pilocarpine treated rats tested during the latency period, i.e., 8–9 days post SE (59).

### 3.3 Social transmission of food preference

Another test capable of measuring context memory is social transmission of food preference, which is used to assess social communication ability. It is often performed to evaluate autism-like characteristics, more rarely to assess memory in rodents. However, studies of social transmission of food preferences in rats have shown that animals are more likely to eat novel food if they smell the food on another rat’s breath and after observing another rat eating that food (60). To our best knowledge, this test was not yet used to assess the associative memory in the pilocarpine model of epilepsy. Thus, this may be an option for epilepsy investigators.

## 4 Recognition memory

### 4.1 Novel object recognition test and its variants

The novel object recognition (or exploration) task and its variants permit recognition of the objects in relation to a defined context. The recognition is possible after adequate training, and it is often launched by a combination of spatial, temporal or emotional inputs (61). Like CFC, memory recognition processes primarily solicit hippocampal, neocortical and BLA circuits (37, 38). Today, the most frequently used test of recognition memory in rodents is the novel object recognition (NOR), sometimes denominated novel object exploration (NOE), object recognition task (ORT) or spontaneous object recognition test (SORT). Its less frequently employed variants are novel object placement, novel object preference and novel context preference tests. A distinct NOR variant is an olfactory discrimination test, which is based on the animals’ ability to detect differences between odors (2 or more in a multiple-choice test). An olfactory discrimination task is based on the association between a sensory stimulus and a food or water reward, and the frequency of correct choice for the stimulus associated with the reward is measured. Olfactory discrimination has an important impact also in NOR and its variants; investigators are used to thoroughly clean with ethanol the apparatus and the objects to remove odor cues before and between their use (62). All the above-mentioned tests have the recognition request in common; experimental animals must recognize the size or the shape of an object; they have to recognize the distance between the objects and walls; they do so in a given context. NOR and its variants overlap with object location test, which is more often used in spatial memory testing (OLT; see below). All object recognition tests are based on the spontaneous behavior of rodents.

The basic NOR task is conducted in an open field arena after habituation (regularly 20–30 min) of animals in the arena the day before. The day of trial, animals are allowed to explore in a first session two identical objects; in a subsequent session, one object is replaced by a different one. The new object should be similar to the original in height and volume, but different in shape and appearance. In NOR test, the objects are placed at an equal distance from the walls of the arena. The outcome measure is the time which the animals spend to explore the familiar and the novel object in the second session. As an alternative, researchers use a discrimination index, that is, the ratio of time spent exploring novel/familiar objects. The most employed time-lapse posed between the two sessions is between 5 min to 2 h. Occasionally, a 24 or 48 h delay can be used to explore the shift between recent and remote recognition memory. Measures are highly influenced by this time interval (63), and, on that base, NOR may be used to discriminate short-term, medium-term and long-term memory by evaluation of the retention interval (i.e., the time the animals maintain memory of sample objects presented during the familiarization phase before the test phase, when one of the familiar objects is replaced by a new one) (64).

The NOR test is relatively easy to perform and is therefore often employed when studying epilepsy comorbidities. An important procedural detail that has been employed in nearly all studies in epilepsy models is to exclude animals that experience a spontaneous recurrent seizure (SRS) during the testing or in the hour preceding it. What is emerging is that pilocarpine or Li-pilocarpine treated rats have a poor performance in NOR and its variants. Researchers report that they spend a similar amount of time exploring both the novel and the familiar object, or even more time with the familiar object, which clearly indicates that epileptic rats are unable to recall a recent recognition memory. Interestingly, similar results are found in the early (at about 2 weeks) post SE phase (65–71), and in the late chronic (1–2 months after SE) phase of the pilocarpine model (66, 67, 72–79). The results obtained during the latency phase are in their greater number parallel to chronic results (80–82), and report similarly less time of novel object exploration and a decreased discrimination index. The above mentioned result are validated in age matched controls. The majority of these studies use a 2 h delay between the training and the assessment phase of the test (65, 66, 68, 71). A few studies apply a 24 h gap between the sessions, but results are similar to those obtained with 2 h delay (67, 72, 81). Only the study of Detour and colleagues, which used the 24 h delay protocol, did not reveal any difference in NOR performance between controls and Li-pilocarpine treated rats tested 5 months after SE (20). In the same paper, however, the authors disclosed a memory decline in the same animals when employing the radial 8 arms maze (20). Another outlier study reported no changes in NOR test with respect to non-epileptic controls at 25 and 40 days after SE (83). Confirming the vast majority of NOR studies, impaired olfactory sensitivity and memory has been described 1 week and 2 months after pilocarpine-induced SE (84).

Whereas the NOR test and its variants strongly converge in identifying memory impairment in chronically epileptic animals after pilocarpine SE, result are more variable in early stages of the disease. In fact, many studies did not report deficits early after SE, i.e., at 3 days (74), at 4 days (83), at 7 days after SE (25, 27, 81, 83). In addition, Bernardi and Barros (25) reported negative results also 2 months after SE utilizing the SORT task. This atypical observation may be due to subtle differences between the NOR and the SORT test.

### 4.2 Novel context preference test

This is an extension of the classic NOR, able to establish if the novelty of objects was important to produce a conditioned increase in environmental preference (85). In the novel context preference test, the animals are given repeated access to familiar and novel objects in different environments and are supposed to display an increase in preference for the paired novelty object-novelty environment (86). The access time to the novel objects is usually at least 10 min. This test may be seen as a natural alternative to FCF, because it gives origin to conditioned association, but not to the fear associated with an environmental cue. However, even if relatively fast and simple, the use of this test is not frequent. Regarding the pilocarpine model, the authors of this review did not find any paper, in which the novel context preference test was used in epileptic animals.

### 4.3 Object location memory test

The object location memory task (OLM or OLT) assesses spatial and reference memory. As NOR and its variants, it is based on the spontaneous tendency of rodents to spend more time exploring a novel object than a familiar object and ability to recognize it even if the object has been relocated (87) but, at the same time, it solicits the working memory employed at maze tasks.

Testing occurs in an open field arena, to which the animals are first habituated. The next day, objects of similar material but different shapes are introduced to the arena. They are spaced roughly equidistant from each other with space in the center, where the animal is put at the start of acquisition (training) phase. In the first trial, the animal is allowed to explore the arena with the objects. In the second trial (often 5 min or 2 h thereafter), the animal again encounters the same objects, except that one or two of them have switched positions. The researchers score the time spent sniffing the objects. The most frequently used version of OLT uses two objects, one of which is replaced in the second trial; a four object variant, with replacement of two, is also often used (86).

OLM task is used for assessing cognitive deficits in epileptic animals, although it has been rarely employed in pilocarpine treated rats. However, pilocarpine rats typically spend less time exploring novel location object with respect to control animals (67, 72, 83). This is observed already at 4 days after SE (83), and persists in late chronic phase (67, 83).

## 5 Working memory

Working memory is a theoretical concept often used synonymously with short-term memory. It is believed to have a limited capacity that can hold information temporarily, to retain what can be important for reasoning and the guidance of decision-making (88). Working memory is sometimes perceived in close relationship with attention, as the attentional control has an additive impact on working memory resources (89). For this reason, some of the tests listed below may be also used for the assessment of attention deficit.

Tests employed to assess working memory include the T-maze and its variant Y-maze, and the 4 or 8-arm radial maze or double H-maze. Different tasks, such as left–right discrimination or forced alternation (to open arms), are used. Maze protocols require food deprivation and may introduce scent cue confounds. In these tests, the working memory is solicited along with spatial learning.

### 5.1 T-maze, Y-maze

T- or Y-mazes are used to assess the decision cognitive ability of rodents. These are T- or Y-letter shaped apparatuses placed horizontally; they can be elevated or wall-enclosed. Animals performing the test are placed at the base of the T/Y and allowed to choose one of the goal arms at the other extremity. It is usually run in two trials of no more than 2 min duration. First, the animal enters one arm. On the second trial, the rodent tends to choose the arm not visited before, thus reflecting memory of the first choice. Such a tendency is called spontaneous alternation and can be reinforced by making the animal hungry and rewarding it with a preferred food in the previously unexplored arm. The number of trials that the animal needs to learn to successfully complete the task can be recorded in the data acquisition phase. Alternatively, the time needed to complete the task can be measured. Whereas two trials (one training and one test trial) may be sufficient for rewarded alternation, spontaneous alternation experiments generally require several training trials. Learning may be made even more difficult by including avoiding entrance into a closed/dark arm, that rodents would instinctively choose. Findings from different studies performed in T- or Y-mazes suggest that both the spontaneous and the rewarded alternation are very sensitive to dysfunction of the hippocampus, although other brain structures may be also involved (31, 90).

T- and Y-mazes have been used to assess the cognitive abilities of epileptic animals using the pilocarpine model. In most studies, a deterioration of spatial working memory was observed. A T-maze test run during the latency phase evidenced a working memory deficit (74, 82, 91, 92). Likewise, a Y-maze tests run in the late chronic phase (40 days post-SE) highlighted a working memory deficit in pilocarpine animals in comparison to controls (93). In contrast with these studies, no difference between pilocarpine and non-epileptic control rats was observed about 1.5 months post SE by Smolensky et al. (49). In these latter study, however, there was a trend to perform worse in pilocarpine rats, and it may be reasonable to expect that this difference could become significant by increasing the number of tested animals.

### 5.2 Eight-arm radial maze, four-arm radial maze

These tests assess the natural ability of rodents to optimize their exploratory strategies. As in the T- or Y-maze tests, both spontaneous and rewarded alternations can be measured. Radial maze can also be used for spatial learning and memory assessment. Besides the classic dry version, an eight-arm radial water maze exists that is designed to evaluate reference and working memory performance while requiring the use extra-maze cues to locate escape platforms (94). Similarly, the double H water-maze is sometimes employed for spatial learning and memory assessment (95). The most employed of the above-mentioned is the 8-arm radial maze. An ordinary 8-arm radial maze consists in 8 equidistantly spaced arms that cross a central open area, which is separated from the arms by trapdoors. The animals are habituated to the apparatus and put off diet before the test to make them starving for food reward (they may lose about 10–15% of body weight). However, standardization of measures that increase motivation to search for the reward is very difficult, and this may increase variability of the results. They are positioned in the center and allowed to freely explore the apparatus, which is initially sprinkled with food all along the arms. In few following days, food is gradually restricted to the end of the arms, so that the animals become familiar with all arms in their whole length. At the time of testing, animals are placed again into the center of the apparatus with all arms filled with food and the doors open; once the animal enters the first arm, all other arms are closed. When the animal finishes all the food in the first arm, it returns to the center and the door of the empty arm behind is closed. The animal is allowed to stay for a few minutes in the center, then all arms open again, and the animal should enter another food filled arm, avoiding the one where it already ate all food. The test finishes when all eight arms are emptied. The test is repeated daily, and the researchers count predominately the number of trials/days needed for animals to successfully complete the task; many evaluate also the errors made by animals during the test execution (e.g., re-entries in empty arms). This test can also differentiate between the working and reference memory in a variant in which only four of the eight arms offer food during the training and the same arms are filled with food during the test. In this variant, two types of errors can be evaluated. A dysfunction of working memory may be identified if an animal re-enters an emptied arm, in which it ate everything before. In analogy, an impairment of the reference memory may be identified if an animal makes an error entering an empty arm, which it should know is empty because it was empty during the training phase.

Radial-arms mazes were used in epileptic rats, predominately the 8-arm radial maze in chronic phase of the disease modeled by pilocarpine (1–5 months’ post SE), documenting a deficit in the working memory. This was usually reported in terms of a significant increase in the number of trials needed to achieve the trial criteria, in terms of the time needed to obtain all pellets or in terms of elevated number of errors (number of wrong entries and re-entries) in epileptic animals as compared with non-epileptic controls (20, 77, 96–100). Similar results were observed with the 4-arm radial maze in pilocarpine and Li-pilocarpine rats 1 month after SE (58). In one of these studies, a positive correlation was observed between the number of SRS and the working memory impairment (98). Working memory deficits were also observed at earlier time-points (15 days’ post SE) in the 8-arm radial maze (93, 101).

As for CFC and recognition memory tests, the results obtained in radial-arms mazes are highly susceptible to interferences associated with epileptogenic events (i.e., behavioral and electroencephalographic seizures) or postictal behavior. Thus, seizure events should represent exclusion criteria. Another potential challenge for epilepsy researchers is the fact that the task requires starving animals, which increase the level of ketone bodies in the brain. This may represent a bias because the ketogenic diet, which can be mimicked by starvation, can be used to treat epilepsy. Indeed, acute or chronic starving of epileptic animals has been reported to reduce the frequency of seizures (102–104). However, none of the above-mentioned studies refers to starvation as an impediment of the experiment.

## 6 Spatial memory tests

Spatial learning and memory is a hippocampal-dependent task (105, 106). The most widely used tests to analyze it in rodents are the Morris water maze, the Barnes maze and the radial eight-arms maze, which was described above; the object location test (OLT), also described above, also offers information on spatial memory.

### 6.1 Morris water maze

The Morris water maze (MWM) test is run in a large circular tank filled with water, which is divided by imaginary lines into four quadrants. In one of these is located a submerged platform, which the animal is expected to find during the trial by using various spatial cues, like objects positioned in fixed places of the tank wall and/or on the walls surrounding the tank. To solve the task, the animal engages in spatial learning and memory, recognizing the place where it is at the start of the task using the cues on the walls.

The most frequently used protocol of MWM foresees an initial (about 2 min) habituation period inside the water tank without platform. In the training phase, the animal is placed into the water at different points of the tank for 4–6 trials of 5–15 min each in a single day. Animals generally find the platform in 1–2 min after the start of the assay. Once the animal finds the platform, it is allowed to stay on it for 10–20 s, then is withdrawn. With advancement of the days of training, it takes progressively less to reach the platform. It may take about 1 week for animals to learn to complete the task under a pre-defined cut-off time, depending on the number of trials per day. Non-epileptic animals usually achieve an optimal performance within 2 consecutive days, when 5–8 trials are applied per day. Five days of MWM cued learning is more than sufficient for intact rats. Latency to escape on platform, distance traveled, swimming speed and number of trials needed to learn to reach the platform within the cut-off conditions, are the parameters used to measure spatial memory (107, 108). It should, however, be considered that there are important sex-differences in many parameters that are routinely assessed through MWM (e.g., male rats are used to travel a greater distance in comparison with females, who in turn search faster than males during habituation and manifest more thigmotactic behavior when exploring the pool) (109).

After the acquisition phase, one may also evaluate the spatial memory recall in a probe test, in which the platform is removed from the pool and the rat is allowed to search for it in a defined time interval, usually 2 min. If the animal recalls correctly the position of the platform, it will spend most of the time swimming in the correct quadrant. Less time spent in the correct quadrant reflects an impairment in spatial memory retention or recall. Probe tests may be also repeated after extra periods of training, thus assessing additional spatial memory characteristics (remote memory recall); the animals after repeated-training are supposed to spend less time and/or to need less trials to learn the task in a supplementary probe test. There is also a variation of the test, in which the quadrant of the platform is changed and the animal’s flexibility and speed to learn the new positions is evaluated (cognitive flexibility testing). The most frequently reported outcome measure of MWM probe test is the time spent in the target quadrant. Other measures are proximity and chance ratio. A specific outcome measure is represented by thigmotactic swimming (i.e., the act of swimming close to the walls of the water maze tank lacking the focus), which may be observed in the acquisition phase but also in probe testing, and which gives a measure of inability to learn the task and engage memory functions. Thigmotactic swimming may be considered an anxiety-like behavior (110).

Given the training in the water and a relatively long time needed for animals to learn the task, this test may not be completely suitable for epileptic animals because, if it experiences a seizure while swimming, it may lose its balance and sink. Spontaneous seizures may be facilitated by the efforts required by the task, especially in chronic epilepsy models. These aspects of the MWM test should be taken into account when preparing the experiment and while interpreting the data. Investigators usually set as exclusion criteria the occurrence of SRS during and within 1 h after the MWM task.

Li-pilocarpine or pilocarpine treated rats show a spatial memory deficit in the MWM test in both cued learning and memory retain. Such findings are shared by many groups, independent of the phase of the disease in the pilocarpine model. The vast majority of the studies that we reviewed report a failure of cued learning (predominately in terms of longer escape latency; 86% of reviewed studies) and spatial memory retrieval (in particular in terms of lower time in or fewer crossings of target quadrant in a probe test; 66% of studies) with respect to intact controls (Table 2). Concerning thigmotactic swimming, the data suggest that animals unable to solve the task have generally a stronger thigmotactic behavior (21, 148). In most of the studies, MWM was run after recognition and maze tests, at the end of the behavioral trials and eventually prior to forced swimming test, because MWM is considered to be more stressful for animals as compared with recognition or mazes but milder than forced swimming (164, 165).

**Table 2:** Use of the Morris water maze in pilocarpine treated rats, at different phases of temporal lobe epilepsy model.

Disease phase	Time post SE	Main finding	Strain	Sex	SE drug dose and route of administration	References
Acute	24 h	↑EL and ↑travel distance	Sprague–Dawley	Male	Pilocarpine (350 mg/kg, i.p.)	(111)
	24 h	↑EL	Sprague–Dawley	Male	LiCl (3 mEq/kg, i.p.) prior to pilocarpine (60 mg/kg s.c.)	(112)
Latency	2 days	↑EL and ↓crossings of TQ	Sprague–Dawley	Male	1% LiCl (3 mg/kg, i.p.) and 1% pilocarpine (30 mg/kg i.p.)	(113)
	1–4 days	↑EL and ↓time in TQ	Wistar	Male	LiCl (127 mg/kg, i.p.), followed 24 h later by pilocarpine (30 mg/kg i.p.)	(114)
	1–5 days	↑EL and ↓time in TQ over 5 days of testing	Sprague–Dawley	Male	LiCl (127 mg/kg, i.p.), followed 24 h later by pilocarpine (30 mg/kg i.p.)	(115)
	1–7 days	↑EL and ↓time in TQ, ↓crossings of TQ the last day of testing	Wistar	Male	LiCl (3 mEq/kg, i.p.) 24.5 h prior to pilocarpine 0.9% (i.p.)	(116)
	3–7 days	↑EL and ↓time in TQ the last (4^th^) day of testing	Sprague–Dawley	Male	Pilocarpine 30 mg/kg (i.p.) than pilocarpine (10 mg/kg, i.p.) every 30 min until the rats developed seizures	(117)
	4–8 days	↑EL and ↓time in TQ over 4 days of testing	Sprague–Dawley	Either	LiCl (3 mEq/kg, i.p.) 20 h prior to pilocarpine (20 mg/kg i.p.)	(118)
	1 week	↑EL and ↓time in TQ	Wistar	Male	LiCl (3 mEq/kg, i.p.) 24 h prior to pilocarpine (30 mg/kg i.p.)	(119)
	1 week	↑distance traveled	Wistar	Male	LiCl (127 mg/kg, i.p.), followed 24 h later by pilocarpine (30 mg/kg i.p.)	(27)
	1 week	↑EL, ↓time in TQ	Sprague–Dawley	Male	Pilocarpine (340 mg/kg, s.c.)	(81)
	1 week	↓time in TQ, ↓crossings of TQ	Sprague–Dawley	Male	LiCl (127 mg/kg, i.p.), followed 18–24 h later by pilocarpine (20 mg/kg i.p.)	(120)
Early chronic	1–2 weeks	↑distance traveled over days 2–3 of testing	Wistar	Male	LiCl (127 mg/kg, i.p.), followed 24 h later by pilocarpine (30 mg/kg i.p.)	(121)	
	10–15 days	↑EL and ↓time in TQ over 5 days of testing	Sprague–Dawley	Male	LiCl (127 mg/kg, i.p.), followed 24 h later by pilocarpine (30 mg/kg i.p.)	(115)	
	12–15 days	↑track length	Wistar	Male	LiCl (127 mg/kg, i.p.), followed 24 h later by pilocarpine (30 mg/kg i.p.)	(49)	
	13 days	↑EL	Sprague–Dawley	Male	LiCl (3 mEq/kg, i.p.) 24.5 h prior to pilocarpine (30 mg/kg, i.p.)	(122)	
	15–19 days	↑EL, ↓time in TQ	Sprague–Dawley	Male	Pilocarpine (280 mg/kg, i.p.)	(65)	
	10, 20 days	↑EL and ↓time in TQ	Sprague–Dawley	Male	LiCl (127 mg/kg, i.p.), followed 18–20 h later by pilocarpine (60 mg/kg i.p.)	(123)	
	2 weeks	↑EL, ↓time in TQ	Wistar	Male	LiCl (127 mg/kg, i.p.), followed 20–24 h later by pilocarpine (30 mg/kg i.p.)	(124)	
	2 weeks	↑EL, ↓time in TQ	Wistar	Male	Pilocarpine (320 mg/kg, i.p.)	(125)	
	2 weeks	↓time in TQ, ↓crossings of TQ	Wistar	Male	LiCl (130 mg/kg, i.p.), followed 24 h later by pilocarpine (50 mg/kg i.p.)	(126)	
	2 weeks	↓time in TQ, ↓crossings of TQ	Sprague–Dawley	Male	Pilocarpine (400 mg/kg, i.p.)	(127)	
	2 weeks	↑EL over days 2–5 of testing	Sprague–Dawley	Male	Pilocarpine (340 mg/kg, i.p.)	(128)	
	2 weeks	↑EL and ↓time in TQ over days 2–5 of testing	Sprague–Dawley	Male	LiCl (180 mg/kg, i.p.), followed 18–20 h later by pilocarpine (30 mg/kg i.p.)	(129)	
	2 weeks	↑EL and ↓time in TQ (day 5 of testing)	Sprague–Dawley	Male	LiCl (127 mg/kg, i.p.), followed 18–20 h later by pilocarpine (30 mg/kg i.p.)	(130)	
	2 weeks	↑EL and ↓time in TQ	Sprague–Dawley	Male	LiCl (3 mg/kg, i.p.), 18 h after pilocarpine (10 mg/kg, i.p.) every 30 min until the rats developed seizures	(131)	
	2 weeks	↑EL	Sprague–Dawley	Male	LiCl (20 mg/kg, i.p.), followed 24 h later by pilocarpine (50 mg/kg i.p.)	(132)	
	2 weeks	↑EL, ↓time in TQ, ↓crossings of TQ over 4 days of testing	Sprague–Dawley	Male	LiCl (127 mg/kg, i.p.), followed 18 h later by pilocarpine (34 mg/kg, i.p.)	(133)	
	2 weeks	↑EL, ↓time in TQ	Sprague–Dawley	Male	LiCl (127 mg/kg, i.p.), followed 18 h later by pilocarpine (34 mg/kg, i.p.)	(134)	
	15 days	↑EL	Sprague–Dawley	Male	LiCl (3 mEq/kg, i.p.) 18 h prior to pilocarpine (60 mg/kg, s.c.)	(135)	
	15–20 days	↑EL over 5 days of testing	Wistar	Male	Pilocarpine (l μL, 2.4 mg/animal, i.h.)	(136)	
	19 days	↓time in TQ	Wistar	Male	LiCl (127 mg/kg, i.p.), followed 20 h later by pilocarpine (60 mg/kg, i.p.)	(137)	
	2–3 weeks	↑EL	Wistar	Male	LiCl (127 mg/kg, i.p.), followed 20–22 h later by pilocarpine (30 mg/kg, i.p.) and an additional 10 mg/kg dose every 30 min afterwards until development of convulsive seizures	(138)	
	3 weeks	↑EL and ↓time in TQ, ↓crossings of TQ	Sprague–Dawley	Male	LiCl (127 mg/kg, i.p.), followed 18–20 h later by pilocarpine (30 mg/kg, i.p.)	(139)	
	22 days	↑EL, ↓time in TQ	Wistar	Male	LiCl (127 mg/kg, i.p.), followed 24 h later by pilocarpine (30 mg/kg, i.p.)	(140)	
	2–4 weeks	↓time in TQ	Sprague–Dawley	Male	Pilocarpine (350 mg/kg, i.p.)	(141)
Late chronic	25–28 days	↑EL	Wistar	Either	LiCl (127 mg/kg, i.p.), followed 24 h later by pilocarpine (40 mg/kg, i.p.)	(75)	
	4 weeks	↑EL over days 2–5 of testing	Wistar	Male	LiCl (127 mg/kg, i.p.), followed 18.5–20.5 h later by pilocarpine (20 mg/kg, i.p.)	(142)	
	4 weeks	↑EL, ↑distance traveled	Wistar	Male	Pilocarpine (340 mg/kg, i.p.)	(143)	
	4 weeks	↑EL and ↓time in TQ	Sprague–Dawley	Male	LiCl (125 mg/kg, i.p.), followed 18–20 h later by pilocarpine (20 mg/kg, i.p.)	(144)	
	4 weeks	↑EL in days 2–5 of testing	Wistar	Male	LiCl (120 mg/kg, i.p.), followed 18 h later by pilocarpine (30 mg/kg, i.p.)	(145)	
	4 weeks	↑EL	Sprague–Dawley	Male	LiCl (3 mEq/kg, i.p.) 24 h prior to pilocarpine (60 mg/kg, i.p.)	(23)	
	4 weeks	↑EL and ↓time in TQ	Sprague–Dawley	Male	LiCl (127 mg/kg, i.p.), followed 18–20 h later by pilocarpine (60 mg/kg, i.p.)	(123)	
	4 weeks	↑EL and ↓time in TQ over 6 days of testing	Sprague–Dawley	Male	LiCl (127 mg/kg, i.p.), followed 18–24 h later by pilocarpine (20 mg/kg, i.p.)	(120)	
	35–42 days	↑EL	Sprague–Dawley	Male	LiCl (127 mg/kg, i.p.), followed 17–20 h later by pilocarpine (30 mg/kg, s.c.)	(146)	
	6 weeks	↑EL and ↓time in TQ	Wistar	Male	LiCl (3 mEq/kg, i.p.) 24 h prior to pilocarpine (45 mg/kg, i.p.)	(147)	
	6 weeks	↑EL and ↓time in TQ	Wistar	Male	LiCl (127 mg/kg, i.p.), followed 19–24 h later by pilocarpine (30 mg/kg, i.p.)	(148)	
	38–45 days	↑EL and ↓time in TQ over 5 days of testing	Wistar	Male	LiCl (127 mg/kg, i.p.) and pilocarpine (60 mg/kg, i.p.)	(149)	
	45 days	↑EL over days 2–5 of testing	Sprague–Dawley	Male	Pilocarpine (340 mg/kg, i.p.)	(150)	
	7 weeks	↑EL and ↓time in TQ, ↓crossings of TQ	Sprague–Dawley	Male	LiCl (127 mg/kg, i.p.), followed 20 h later by pilocarpine (50 mg/kg, i.p.)	(151)	
	54 days	↑EL	Wistar	Male	Pilocarpine (350 mg/kg, i.p.)	(152)	
	52–59 days	↑EL, ↓time in TQ	Sprague–Dawley	Male	LiCl (127 mg/kg, i.p.), followed 18 h later by pilocarpine (50 mg/kg, i.p.)	(153)	
	55–59 days	↑EL over 5 days of testing	Sprague–Dawley	Male	LiCl (127 mg/kg, i.p.), followed 24 h later by pilocarpine (20 mg/kg, i.p.)	(154)	
	57–61 days	↑EL and ↓time in TQ	Wistar	Male	LiCl (3 mEq/kg, i.p.) 20 h prior to pilocarpine (35 mg/kg, i.p.)	(73)	
	7–8 weeks	↑EL and ↓time in TQ, ↓crossings of TQ	Sprague–Dawley	N/A	LiCl (127 mg/kg, i.p.), followed 16–18 h later by pilocarpine (30 mg/kg, i.p.)	(155)	
	8 weeks	↑EL and ↓time in TQ	Sprague–Dawley and Wistar	Male	LiCl (127 mg/kg, i.p.), followed 12–24 h later by pilocarpine (10 mg/kg, i.p.) four doses every 30 min	(21)	
	60 days	↑EL and ↓time in TQ	Wistar	Male	Pilocarpine (320 mg/kg, i.p.)	(52)	
	2 months	↑EL at day 2 of probe trial	Wistar	Male	LiCl (127 mg/kg, i.p.), followed 24 h later by pilocarpine (20–40 mg/kg, i.p.)	(156)	
	61–65 days	↑EL and ↓crossings of TQ	Wistar	Male	LiCl (3 mmol/kg, i.p.), followed 18.5 h later by pilocarpine (35 mg/kg, i.p.)	(157)	
	65 days	↑EL	Sprague–Dawley	Male	LiCl (3 mEq/kg, i.p.) 18 h prior to pilocarpine (60 mg/kg, s.c.)	(135)	
	60–66 days	↑EL and ↓time in TQ over 5 days of testing	Sprague–Dawley	Male	LiCl (127 mg/kg, i.p.), followed 18 h later by pilocarpine (50 mg/kg, i.p.)	(78)	
	70–90 days	↑EL	Wistar	Male	LiCl (127 mg/kg, i.p.), followed 25 h later by pilocarpine (10 mg/kg, i.p.) four doses every 30 min	(55)	
	60–66 and 90–96 days	↑EL and ↓time in TQ in 2 probe trials	Wistar	Male	Pilocarpine (350 mg/kg, i.p.)	(158)
	30, 50, and 100 days	↑distance traveled and ↓time in TQ	Wistar	Male	Pilocarpine (340 mg/kg, i.p.)	(159)
	81 days	↑EL and ↓time in TQ	Sprague–Dawley	Male	LiCl (127 mg/kg, i.p.), followed 20.5 h later by pilocarpine (25 mg/kg, i.p.)	(95)
	3 months	↑EL and ↓time in TQ, over 4 days of testing	Sprague–Dawley	Male	LiCl (127 mg/kg, i.p.), followed 24.5 h later by pilocarpine (40 mg/kg, i.p.)	(160)
	3 months	↑EL, ↑distance traveled and ↓time in TQ	Wistar	Male	Pilocarpine (320 mg/kg, i.p.)	(96)
	3 months	↑EL and ↑number of failures	Wistar	Male	LiCl (3 mmol/kg, i.p.), followed 24 h later by pilocarpine (40 mg/kg, i.p.)	(161)
	3 months	↑EL	Wistar	Male	LiCl (3 mEq/kg, i.p.), followed 24 h later by pilocarpine (30 mg/kg, s.c.)	(50)
	104–110 days	↑EL and ↓crossings of TQ over 5 days of testing	Wistar	Male	Pilocarpine (280 mg/kg, i.p.)	(79)
	4 months	↓time in TQ	Wistar	Male	LiCl (127 mg/kg, i.p.), followed 24 h later by pilocarpine (25 mg/kg, i.p.)	(162)
	4 months	↑EL, ↑distance traveled and ↓time in TQ	Wistar	Male	Pilocarpine (320 mg/kg, i.p.)	(163)

The main finding refers to the principal finding regard to the control group observed in pilocarpine or Li-pilocarpine treated animals and is reported in terms of escape latency (EL) and time of crossings of target quadrant (TQ). Drug dose and route of administration (whenever available) refer to pilocarpine or Li-pilocarpine combination (LiCl—lithium chloride; pilocarpine—pilocarpine hydrochloride) used to induce status epilepticus (SE) in a given study. N/A—information not available.

### 6.2 Barnes maze test

The Barnes maze (BM) assay is one of the three mostly employed assays to test spatial learning and memory (with the MWM and radial 8-arms maze). In the Barnes (dry land) maze, no strong aversive stimulus (like swimming in MWM) or food deprivation for reinforcement (like in the radial 8-arms maze) are used (166–168). Instead, a natural preference of rodents for the dark and closed places is exploited.

The apparatus of BM consists of a circular plane with 18–20 circular holes arranged along its entire circumference; the plane is bright light illuminated; under one of the holes is placed a cage, the only shelter available; animals should find the way to this hole during the task using spatial cues (visual reference points placed around the circular plane). Usually, it takes about 4 to 7 training repetitions to teach the animal to proceed quickly in a straight line toward the hole that hides the drop box (169). BM performance is not associated with significant stress, that may instead compromise other spatial memory tests (166). However, the absence of a strong aversive stimulus may reduce the motivation to complete the operation. If it is the case, a food reinforcement may be considered (170).

The test is run very much like MWM. Experimenters let the animal explore the maze during the habituation; then allow it to learn the position of an escape hole during the training trials; finally, spatial memory retention is tested by putting the animal again into the maze with the escape hole blocked. One recent variant of BM was used to test social interaction and may be classifies as a test assessing contextual memory; in such extension of BM rats (in the role of observers) improve their learning by observing the behavior of other rats (models) that had already acquired the task (171).

The parameters used to assess the classic BM include the number of trials needed to learn the position of the escape hole during the acquisition phase (i.e., the training) and the time spent in the vicinity of the escape hole in the probe test. The BM test has been used less frequently than MWM in epileptic rats, but seems to become progressively more popular (increasing number of studies published in the last decade). One of the reasons why BM is gaining popularity over MWM is that it is less stressful for rodents, as indicated by the fact that, contrary to MWM, it does not increase corticosterone levels (166, 172–174).

The BM test was successfully applied in the pilocarpine model. In all studies, the epileptic rats had significantly increased latency to find the hidden escape box when compared to matched controls. The results are consistent along the course of disease; an impairment in spatial navigation learning and memory was observed both in early chronic phase (69, 71, 80, 175) and late chronic phase (156, 176, 177) of the temporal lobe epilepsy.

### 6.3 Hole board task

The hole-board may be seen as a variant of the BM test. It consists of a small square arena with an extractable platform as floor, which has a set of equally spaced circular holes on its surface. Typically, it has an even number of holes (very frequent is 4-hole-board). Similarly, to BM, it allows the assessment of long-term spatial memory in rodents without the employment of water or food restriction, painful stimuli (electrical shocks) or any other aversive condition. In the 4-hole-board variant, holes are equidistant (one for each of the resulting quadrants). Animals put in the arena spontaneously approach the holes and explore them. If they are re-exposed to the hole-board, they lose interest in the novelty of the holes, unless researchers use a food reinforcement. Animals with an intact long-term memory show a reduction of the frequency of exploring the holes by nose-poking, or do not make errors when searching for the food. Usually, the total number of nose-pokes or the number of correct nose-pokes in food reward variants are counted and used as an index of long-term spatial memory. These numbers are stable across the days of testing in spatial memory of compromised animals.

We identified only one work in which hole board task was employed for assessment of spatial memory in pilocarpine rats. Patra et al. (178) report a significant spatial memory deficit at about 4 months after SE.

## 7 Other memory tests

### 7.1 The lateralized reaction time task

The lateralized reaction time task (LRTT) is mentioned because it is sometimes used to evaluate attention in pilocarpine treated animals. LRTT measures the basic cognitive processes of perception and response execution. It requires that animals make one specific response (e.g., a spacebar press) whenever a stimulus (e.g., a shape) appears. A laterally driven stimulus is delivered unexpectedly which permits the investigator to differentiate between stimulus-driven and controlled attention (179). LRTT can be digitized, such that the stimuli appear on the screen. Typically, there is only one type of stimulus, and it repeats throughout the experiment. The main utilized LRTT outcome measure is the speed of responding. This test was employed in epileptic rats by Pineda et al. (180) at about 2.5 months after SE, documenting a reduced attention in pilocarpine treated animals.

### 7.2 Social interaction tests

Social recognition memory tests and their variants resident intruder tests reflect the ability of social animals to recognize and remember individuals of the same species, preferring social interaction over no social interaction and social novelty (i.e., they prefer interaction with a novel animal over a familiar animal) (181). The most popular tests of this kind are the two-trial social recognition test, the habituation-dishabituation paradigm test and discrimination paradigm test, all evaluating the time of interaction (i.e., olfactory investigation) of familiar and non-familiar subject. Rats with unimpaired social recognition memory spend less time investigating familiar individuals, compared to novel individual (182). For example, in the two-trial social recognition test the tested animal at first encounters another animal, then, in the following trial, encounters either the same animal (familiar) or another unknown animal (non-familiar); the measured parameter is reduction in time of investigation of the familiar animal, which reflects the establishment of social recognition memory (183). The habituation-dishabituation paradigm instead consists of five trials, in which the familiar animal is presented to the tested one for the first four trials and the non-familiar animal is introduced in the last trial. Similar to the two-trial social recognition test, a reduction in investigation time with the familiar animal may be observed over the trials, while investigation time increases with the non-familiar rat (184). With the discrimination paradigm the interaction occurs between the tested animal and the familiar and non-familiar ones simultaneously; again, the interaction time with each reflects social memory (185).

A few studies have revealed compromised social recognition memory in epileptic animals. A prolonged interaction time with the familiar animal was observed in epileptic rats at 8 and 56 days post SE (84). Another study reported impaired social memory in epileptic rats based on reduced time spent sniffing and grooming the non-familiar animal in comparison with non-epileptic animal (49). Likewise, a reduced motivation for social contact in terms of reduced interaction with non-familiar rats was reported in epileptic animals at 9 weeks post SE (22).

### 7.3 The step-down avoidance task

The Step-Down Avoidance Task (also called light/dark box test) is used for measuring aversive learning and memory, whose presence is observed by behavioral responses following an experience. The test is usually run as a one-trial task combining fear conditioning with an instrumental response, e.g., the active choice of an animal to avoid entering the dark compartment associated with an aversive event (186). The aversive stimulus is most frequently a foot-shock. Like in social interaction tasks, the step-down avoidance has been rarely employed in epileptic animals. However, shorter latency to step into the chamber with electric shock delivery has been reported in pilocarpine rats in the chronic period, 1 or 2 months after SE (75, 79, 187, 188), which indicates learning and memory impairment.

## 8 Limitations and recommendations

The behavioral tests conducted to access cognitive comorbidities in the rat pilocarpine model often face limitations inherent to the epileptic phenotype (3). The most important of them is the fact that, in chronic phase, spontaneous and recurrent seizures occur unpredictably, which could be a confounding factor for behavioral testing. Seizures occurring shortly before or during the test would affect the animal performance and the results. Video monitoring before and during testing could allow the exclusion of such cases (189). In addition, olfactory deficits (84) and hyperactivity (180) have been reported in pilocarpine rats. This could be a bias in tests requiring motor or odor intact functions, such as the maze-based tests and social interaction tests, respectively. In addition, the animals sex should always be reported and took in consideration when designing and analyzing results, because some behavioral tests, such as CFC (190) or MWM (109), have sex specific outcomes. The fear response measured in CFC tests is considerably lower during proestrus, when levels of the sex hormones estradiol and progesterone are high (190). Similarly, several variables measured routinely in MWM have been shown to vary between sexes (e.g., females search slightly faster than males during habituation, travel lower distances during learning or spend less time than males in the pool’s center over the test days) (109). Not only males may be different from females, but also distinct effects in the above mentioned behavioral tests may be found in cycling females during different hormonal phases. This is the main reason why the vast majority of studies reviewed in this work used exclusively males.

Some of these limitations may be overcome by following published recommendations or guidelines. General recommendations which should be followed in any research work involving animal research are described in the ARRIVE guidelines (Animal Research: Reporting of *In Vivo* Experiments) (191). Moreover, the Task Force of the International League Against Epilepsy (ILAE) and the American Epilepsy Society (AES) recently developed a guidance that includes Common Data Elements (CDEs) (192), which describes the different types of assessments and highlights the importance of rigorous data collection and transparent reporting in epilepsy. Tests are divided into 7 categories to examine syndrome-level behavioral dysfunctions, including learning and memory deficits. Guidelines discuss the complexities, limitations, and biases associated with behavioral testing, especially when performed on animals with epilepsy (189).

## 9 Conclusion

In sum, published data provide clear evidence of memory impairment in the pilocarpine model. The combination of results from different studies is often difficult because of variability in methodological standards, number of animals employed, and outcome measures. The use of a more rigorous and reproducible methodology following the ILAE-AES recommendations (192) would certainly favor a further advancement of the field. Nonetheless, a strong evidence emerges that cognitive impairment begins to appear in the pilocarpine model during the latency phase (i.e., before occurrence of SRSs), becoming intense and affecting different types of memory in the chronic phase, thus mimicking the cognitive co-morbidity profile observed in human temporal lobe epilepsy (193, 194). Altogether, this review supports the value of the rat pilocarpine model not only for modeling spontaneous seizures but also temporal lobe epilepsy-associated cognitive comorbidities.

## Data availability statement

The original contributions presented in the study are included in the article/supplementary material, further inquiries can be directed to the corresponding author.

## Author contributions

AG: Writing – original draft, Writing – review & editing. PP: Writing – original draft, Writing – review & editing. FL: Writing – original draft, Writing – review & editing. LA: Writing – original draft, Writing – review & editing. MSi: Funding acquisition, Writing – original draft, Writing – review & editing. MSo: Conceptualization, Investigation, Methodology, Supervision, Writing – original draft, Writing – review & editing.
